# Supplementary material for: Interpatient Heterogeneity in Drug Response and Protein Biomarker Expression of Recurrent Ovarian Cancer
Source: Cancers (Basel). 2022 May 3;14(9):2279. doi: 10.3390/cancers14092279 (PMC9103312; doi:10.3390/cancers14092279)
Supplement: Supplementary file 1 [file cancers-14-02279-s001.zip › cancers-1667043-supplementary.pdf]

Supplemental Material

Table S1. Antibodies and detection systems.

Antigen	Antibody/ Clone	Species	Isotype	Working concentration µg/ml	Kit	Source
EGF-R	H11	m	IgG1	2.94	-	Dako
Her2/neu	4B5	r	IgG1	1.5	-	Novocastra
ERα	1D5	m	IgG1	2.5	+	Dako
PR	PgR636	m	IgG1	2.5	+	Dako
IGF1R	24-31	m	IgG1	4.0	+	Dako
HGFR	SP44	r	IgG1	2.12	-	Biomol
EpCAM	Ber-EP4	m	IgG1	2.5	-	Dako
$\alpha 2\beta 1$	BHA2.1	m	IgG1	2.5	-	Millipore
$\alpha V\beta 3$	LM609	m	IgG1	5.0	-	Millipore
MUC1	Ma55.2	m	IgG1	0.5	-	Novocastra
CD44v6	VFF-18	m	IgG1	1.0	-	eBioscience
HSP90	AC88	m	IgG1	10	+	Abcam
Ribosomal protein S6	rpS6 poly	r	poly-clonal	0.24	-	Cell Signaling
PD-L1	MIH1	m	IgG1	10	+	Affymetrix
**Positive control**						
Pan Cytokeratin	KL-1	m	IgG1	0.32	-	Zytomed Systems
**Negative controls**						
	MOPC-21	m	IgG1	2.5, 5.0	-	Sigma-Aldrich
	MOPC-21	m	IgG1	10	+	
	DA1E	r	IgG1	2.12	-	Cell Signaling
	Rabbit serum	r	Poly-clonal	0.24	-	Dako
**Biotinylated secondary antibodies and detection system**						
	315-065-048	r anti m biotin	IgG+IgM	0.75	-	Dianova
	111-065-144	g anti r biotin	IgG	7	-	Dianova
	016-030-084	POX-strept avidin	-	1	-	Dianova

Legend: m, mouse; r, rabbit; g, goat; POX-streptavidin, peroxidase-conjugated streptavidin.

Table S2. Mean cell viability [%] +/- SD of of recurrent ovarian cancer after therapy analyzed in the ovarian cancer spheroid model

Patient	Localisation	Therapies										
		Carboplatin	Carboplatin + Paclitaxel	Carboplatin + Gemcitabine	Carboplatin + PLD	Paclitaxel	Gemcitabine	PLD	Topotecan	Etoposide	Treosulfan	Vinorelbine
1	Peritoneum 1	58,45 ± 5,34	55,98 ± 2,86	48,45 ± 4,61	49,14 ± 7,01	94,39 ± 13,70	82,88 ± 9,56	77,05 ± 5,90	69,72 ± 18,60	n.t.	26,67 ± 2,76	63,05 ± 11,56
2	Peritoneum	75,09 ± 4,18	55,36 ± 1,70	67,82 ± 8,33	n.t.	77,33 ± 5,25	108,02 ± 4,17	n.t.	64,10 ± 3,35	n.t.	70,80 ± 4,53	72,17 ± 7,86
3	Peritoneum	73,35 ± 5,21	50,20 ± 1,45	58,43 ± 9,92	n.t.	80,19 ± 17,07	83,30 ± 20,02	n.t.	82,24 ± 12,56	n.t.	45,44 ± 7,22	72,56 ± 8,64
4	Peritoneum	67,48 ± 6,15	41,97 ± 0,81	40,12 ± 5,25	n.t.	67,48 ± 1,43	99,95 ± 6,41	n.t.	54,71 ± 4,84	n.t.	28,08 ± 4,39	47,88 ± 4,62
5	Peritoneum 1	61,06 ± 7,78	38,02 ± 5,06	29,16 ± 5,49	n.t.	85,35 ± 5,27	110,36 ± 15,67	n.t.	25,30 ± 2,99	n.t.	3,62 ± 0,50	32,97 ± 2,58
	Peritoneum 2	55,81 ± 6,79	38,81 ± 6,11	16,08 ± 1,25	n.t.	95,25 ± 4,45	91,86 ± 8,92	n.t.	47,15 ± 7,78	n.t.	6,51 ± 2,29	51,28 ± 1,72
6	Peritoneum	57,32 ± 0,63	37,65 ± 3,17	27,06 ± 0,59	n.t.	77,60 ± 5,05	100,47 ± 5,38	n.t.	27,16 ± 1,20	n.t.	1,90 ± 0,26	22,37 ± 0,91
7	Peritoneum	n.t.	36,81 ± 4,74	26,07 ± 1,32	n.t.	n.t.	n.t.	n.t.	n.t.	n.t.	51,07 ± 5,18	n.t.
8	Peritoneum 1	42,15 ± 3,86	34,42 ± 3,67	22,17 ± 1,83	27,36 ± 2,53	95,26 ± 3,72	77,67 ± 4,26	90,73 ± 5,65	84,83 ± 0,74	75,17 ± 8,84	9,18 ± 1,04	57,54 ± 4,14
	Peritoneum 2	41,04 ± 3,17	32,41 ± 1,99	19,02 ± 1,35	29,74 ± 1,67	71,08 ± 8,10	55,06 ± 5,66	70,81 ± 5,33	77,92 ± 7,48	43,88 ± 6,29	6,98 ± 1,56	44,31 ± 6,34
9	Peritoneum 1	16,29 ± 1,64	19,40 ± 1,12	14,40 ± 1,74	19,73 ± 9,68	84,06 ± 6,76	86,59 ± 4,54	84,61 ± 0,86	48,86 ± 7,65	n.t.	17,29 ± 4,23	65,04 ± 0,17
	Peritoneum 2	32,72 ± 3,05	31,62 ± 1,90	12,27 ± 1,38	25,49 ± 1,83	76,21 ± 3,35	71,56 ± 0,92	74,68 ± 4,02	41,51 ± 3,28	n.t.	11,40 ± 4,32	64,04 ± 5,70
10	Peritoneum 1	39,10 ± 1,63	22,10 ± 4,20	19,20 ± 3,23	28,30 ± 1,15	73,71 ± 0,96	107,05 ± 18,69	61,75 ± 6,11	51,33 ± 7,79	55,57 ± 3,51	14,02 ± 1,40	34,90 ± 4,35
	Peritoneum 2	58,15 ± 5,19	29,97 ± 4,41	41,25 ± 7,41	57,20 ± 5,94	53,28 ± 4,03	102,99 ± 12,72	62,36 ± 7,03	55,56 ± 7,54	39,21 ± 14,19	22,81 ± 4,22	46,11 ± 7,65
	Omentum	36,22 ± 3,23	22,54 ± 1,14	9,63 ± 2,09	31,06 ± 4,17	94,73 ± 10,90	76,23 ± 6,13	77,71 ± 0,30	35,69 ± 4,87	63,79 ± 1,55	5,77 ± 2,07	14,76 ± 1,93
11	Peritoneum	28,79 ± 3,07	21,95 ± 3,31	15,23 ± 2,23	n.t.	62,48 ± 3,32	59,96 ± 8,31	n.t.	56,24 ± 3,69	n.t.	14,96 ± 1,60	63,06 ± 4,37
12	Intraparenchymatous tissue, liver	36,11 ± 3,81	17,23 ± 1,01	6,23 ± 0,76	29,17 ± 5,66	70,02 ± 6,03	60,70 ± 5,41	75,48 ± 5,18	25,38 ± 2,91	59,22 ± 5,43	1,70 ± 0,34	11,52 ± 1,27
13	Metastasis (Liver)	28,07 ± 1,46	14,77 ± 0,55	6,85 ± 0,74	19,96 ± 0,79	78,81 ± 2,83	78,76 ± 4,65	73,88 ± 2,51	31,99 ± 1,99	n.t.	12,48 ± 2,58	22,65 ± 4,83
14	Peritoneum 1	16,86 ± 1,28	9,15 ± 0,99	3,62 ± 0,75	n.t.	65,81 ± 3,22	88,59 ± 8,37	n.t.	34,80 ± 3,19	n.t.	5,30 ± 0,77	13,82 ± 2,40
	Peritoneum 2	17,81 ± 1,26	14,36 ± 2,71	6,40 ± 1,58	n.t.	55,99 ± 8,91	97,07 ± 17,66	n.t.	23,29 ± 4,48	n.t.	5,55 ± 1,40	27,52 ± 2,54
15	Peritoneum	22,76 ± 1,76	14,24 ± 0,17	8,48 ± 0,65	16,10 ± 0,34	83,74 ± 3,23	36,68 ± 4,08	92,89 ± 7,84	42,89 ± 3,13	40,32 ± 3,10	50,21 ± 1,65	114,31 ± 1,69
16	Peritoneum	8,67 ± 0,44	5,94 ± 0,44	3,66 ± 0,07	n.t.	52,19 ± 4,21	65,79 ± 1,63	n.t.	25,43 ± 0,10	n.t.	7,43 ± 0,58	33,39 ± 0,11
	Omentum minor	19,11 ± 0,22	12,82 ± 0,75	7,34 ± 1,02	n.t.	82,18 ± 4,23	72,63 ± 2,92	n.t.	36,01 ± 1,63	n.t.	5,523 ± 0,20	37,56 ± 2,68
17	Peritoneum	18,10 ± 0,70	11,46 ± 0,93	9,98 ± 0,49	13,55 ± 0,36	81,24 ± 7,80	99,01 ± 12,95	78,79 ± 0,25	38,18 ± 0,78	85,97 ± 1,29	13,79 ± 1,66	48,82 ± 1,70
18	Peritoneum	13,51 ± 0,85	7,24 ± 0,16	4,43 ± 0,13	n.t.	66,15 ± 5,80	74,42 ± 2,23	n.t.	37,75 ± 5,47	40,94 ± 7,79	17,45 ± 2,73	47,57 ± 5,29
19	Peritoneum	15,63 ± 4,19	7,18 ± 0,17	5,99 ± 0,42	6,02 ± 1,51	88,17 ± 11,41	62,41 ± 7,44	61,25 ± 1,22	76,32 ± 2,52	66,41 ± 5,49	5,67 ± 0,64	47,25 ± 5,04
20	Metastasis (Liver)	9,44 ± 0,29	5,91 ± 0,20	3,64 ± 0,83	n.t.	72,21 ± 1,56	75,08 ± 3,09	n.t.	68,07 ± 1,10	n.t.	9,77 ± 1,44	33,11 ± 5,55
21	Peritoneum	5,88 ± 0,74	4,66 ± 0,53	4,19 ± 1,22	n.t.	52,99 ± 2,48	75,67 ± 15,45	n.t.	40,37 ± 4,58	n.t.	35,03 ± 4,14	40,19 ± 2,70
22	Peritoneum 1	14,81 ± 1,12	3,69 ± 0,57	1,35 ± 0,20	4,66 ± 0,58	78,45 ± 2,39	52,46 ± 4,79	73,04 ± 3,51	31,76 ± 1,21	63,16 ± 0,68	2,09 ± 0,44	11,98 ± 0,64
	Peritoneum 2	9,90 ± 1,36	3,73 ± 0,36	3,85 ± 0,49	3,88 ± 0,29	71,61 ± 3,69	69,97 ± 9,97	73,85 ± 2,38	26,89 ± 4,38	75,33 ± 1,74	4,14 ± 0,61	33,60 ± 0,57

n.t. not tested, PLD PEGylated liposomal doxorubicin, SD standard deviation, Peritoneum 1 and 2 were prepared from different locations in the peritoneal cavity

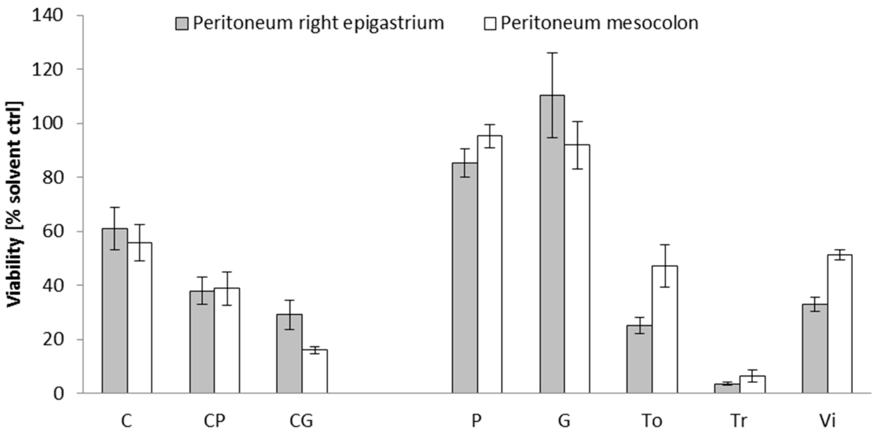

(a) Patient 5

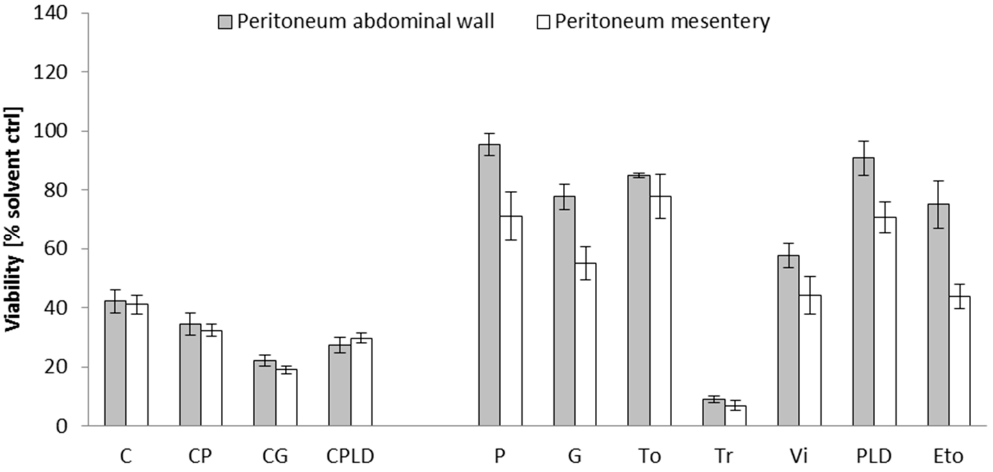

(b) Patient 8

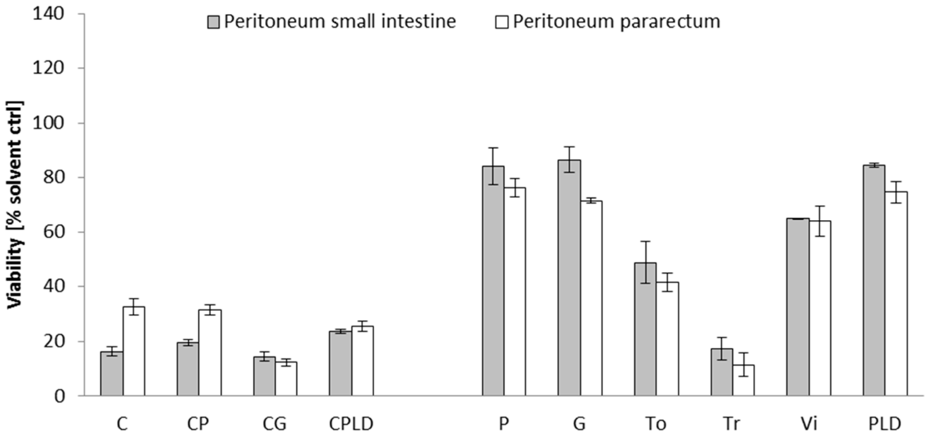

(c) Patient 9

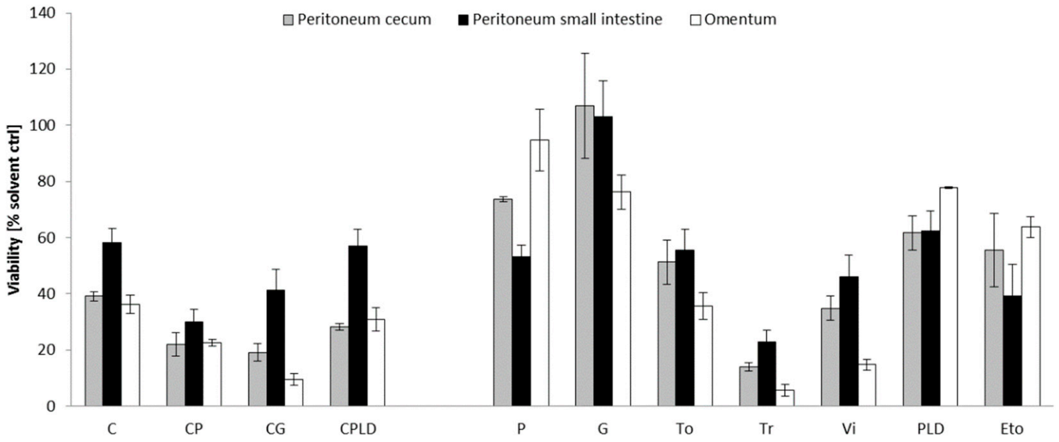

(d) Patient 10

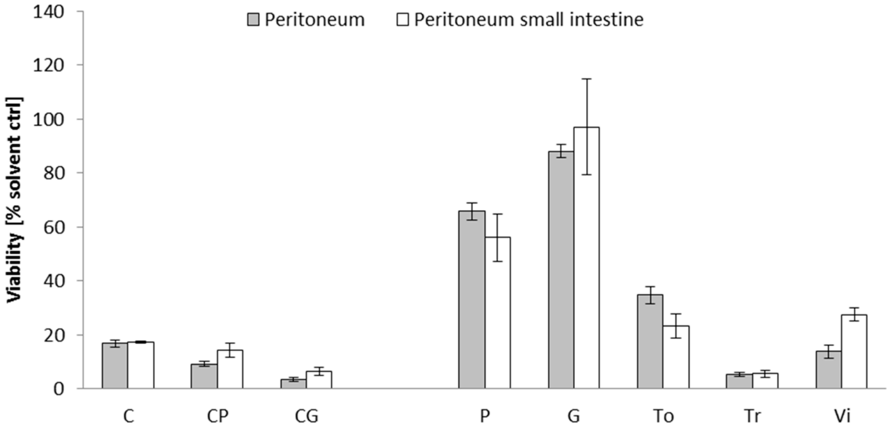

(e) Patient 14

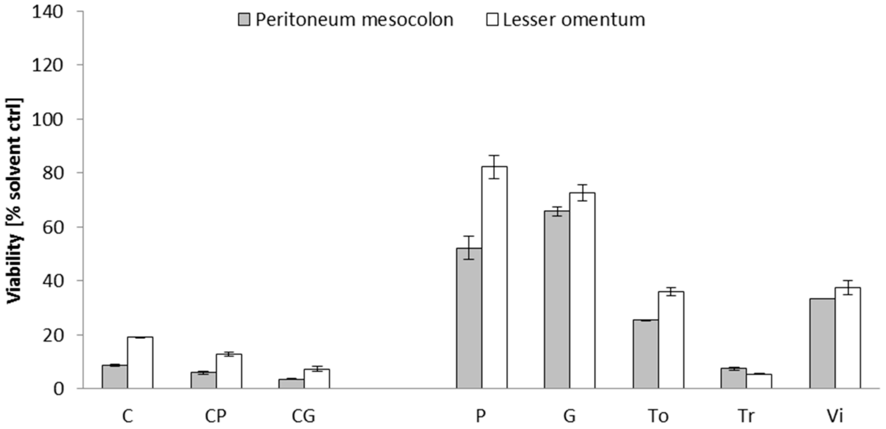

(f) Patient 16

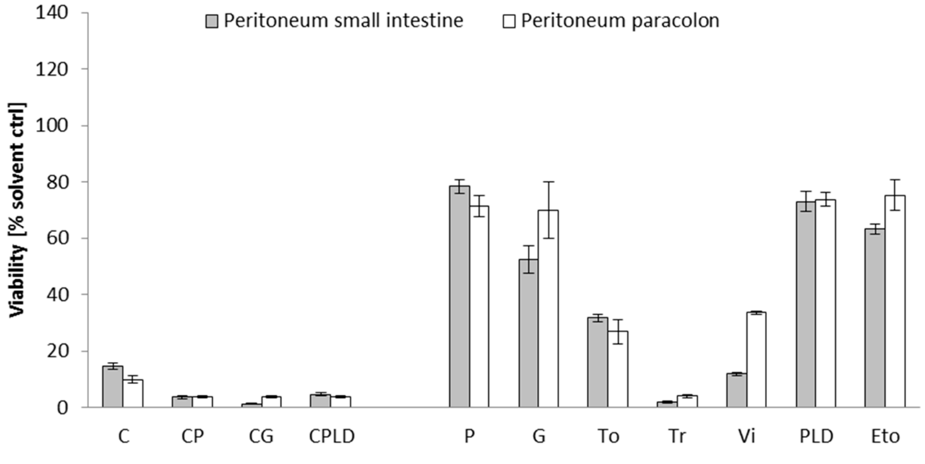

(g) Patient 22

Figure S1. Comparison of the treatment impact between autologous tumor samples.

Figure S2:

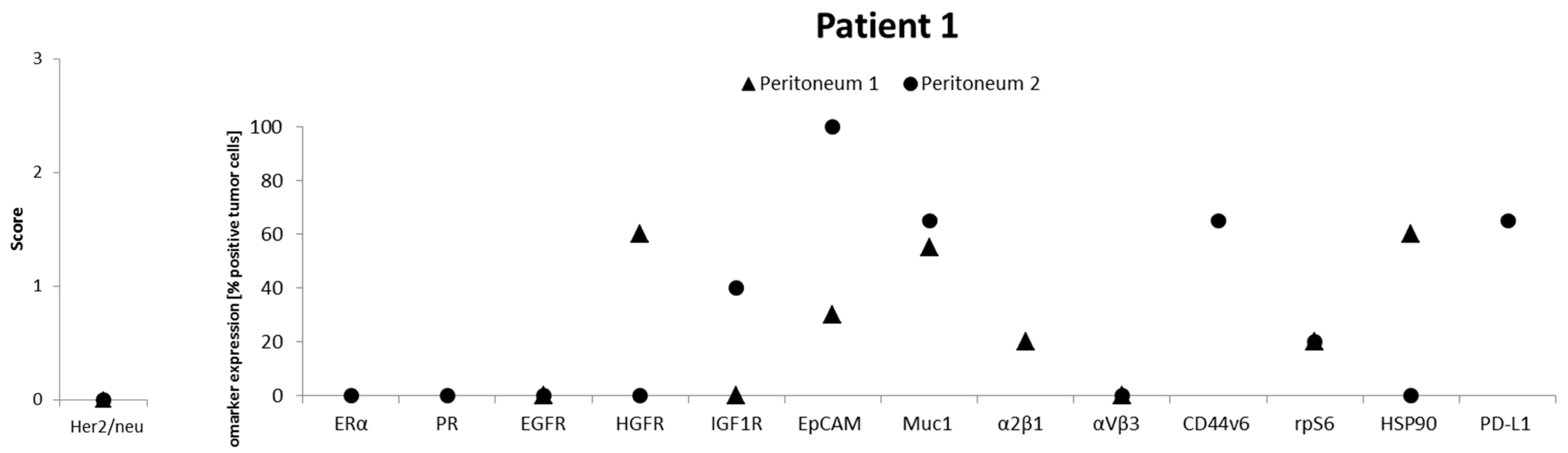

Patient 1
Peritoneum 1
Peritoneum 2
Score
3
2
1
0
Her2/neu
omarker expression [% positive tumor cells]
100
80
60
40
20
0
ERα
PR
EGFR
HGFR
IGF1R
EpCAM
Muc1
α2β1
αVβ3
CD44v6
rpS6
HSP90
PD-L1

(a)

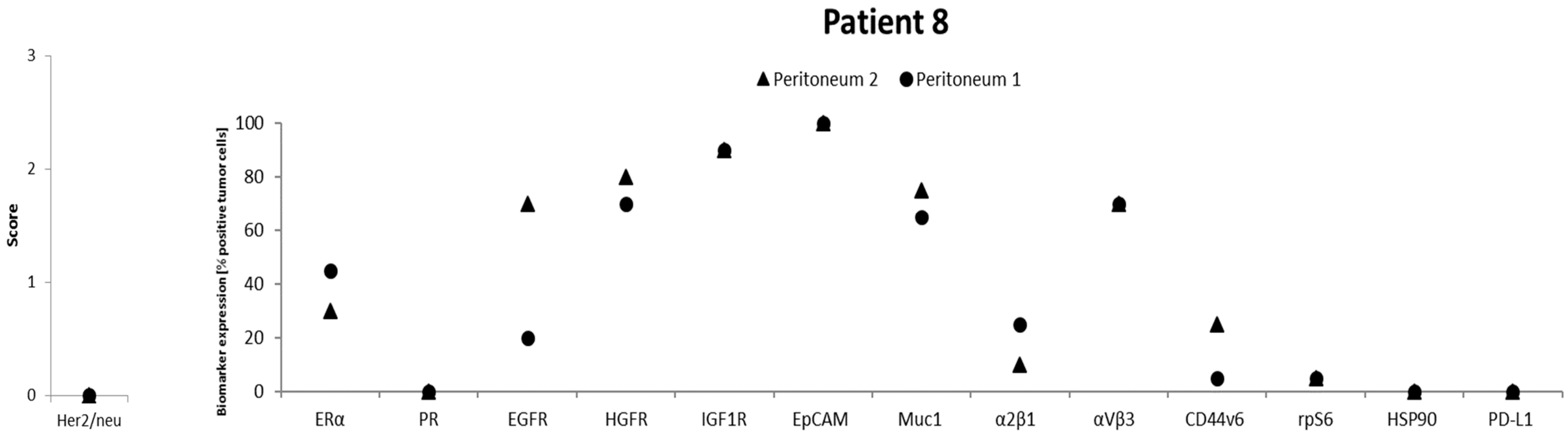

Patient 8
Peritoneum 2
Peritoneum 1
Score
3
2
1
0
Her2/neu
Biomarker expression [% positive tumor cells]
100
80
60
40
20
0
ERα
PR
EGFR
HGFR
IGF1R
EpCAM
Muc1
α2β1
αVβ3
CD44v6
rpS6
HSP90
PD-L1

(b)

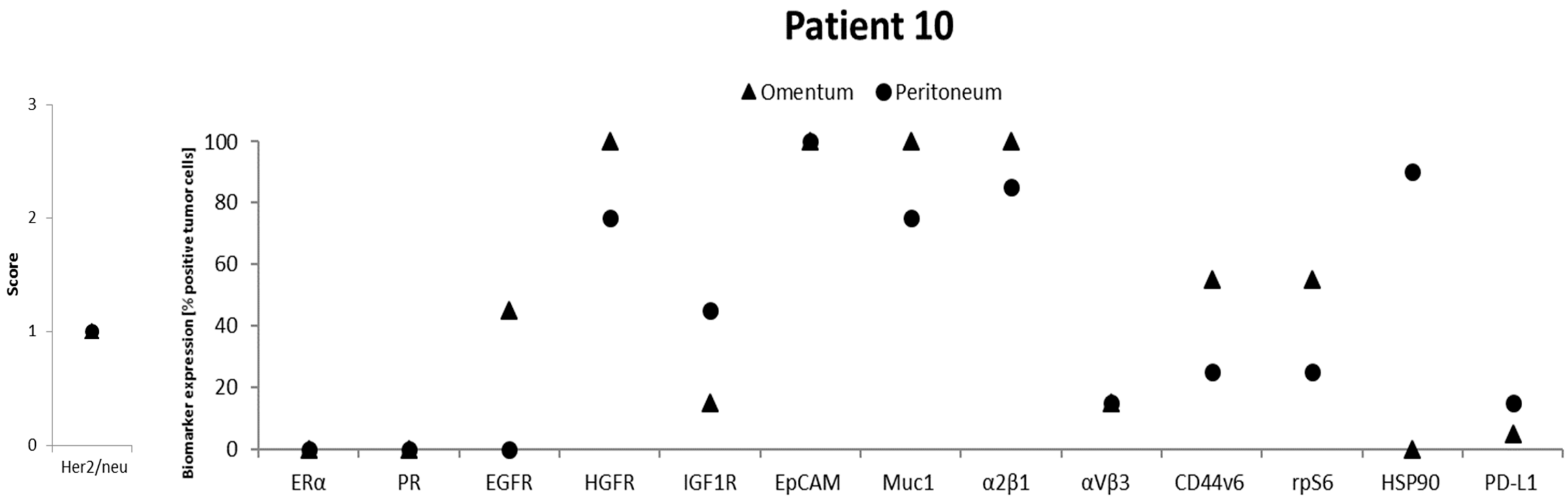
Patient 10
Omentum
Peritoneum
Score
Her2/neu
Biomarker expression [% positive tumor cells]
ERα
PR
EGFR
HGFR
IGF1R
EpCAM
Muc1
α2β1
αVβ3
CD44v6
rpS6
HSP90
PD-L1

(c)

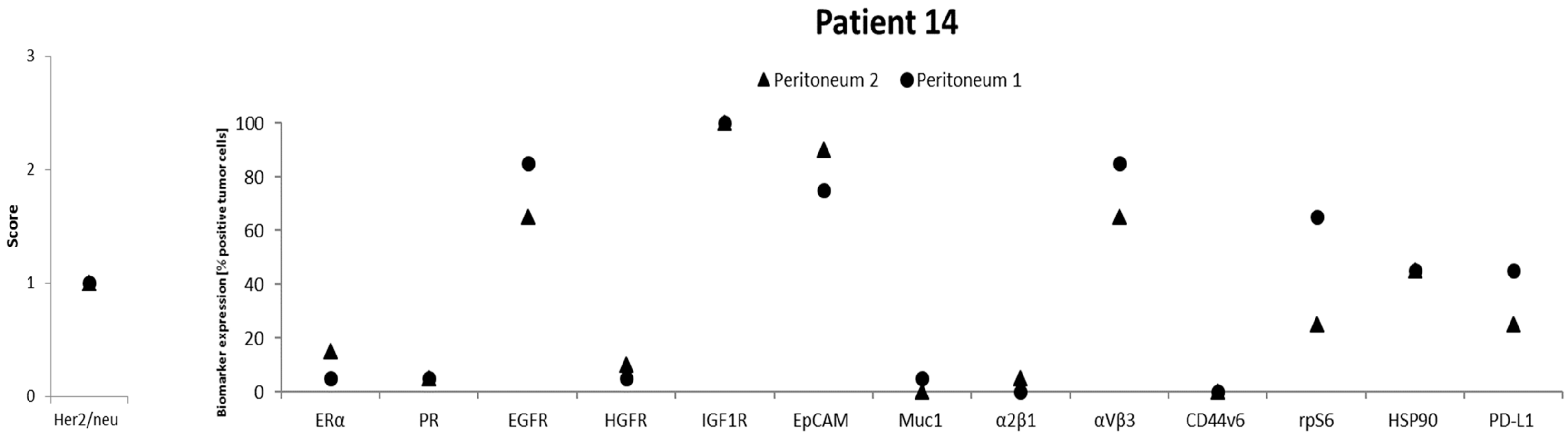
Patient 14
Peritoneum 2
Peritoneum 1
Score
Her2/neu
Biomarker expression [% positive tumor cells]
ERα
PR
EGFR
HGFR
IGF1R
EpCAM
Muc1
α2β1
αVβ3
CD44v6
rpS6
HSP90
PD-L1

(d)

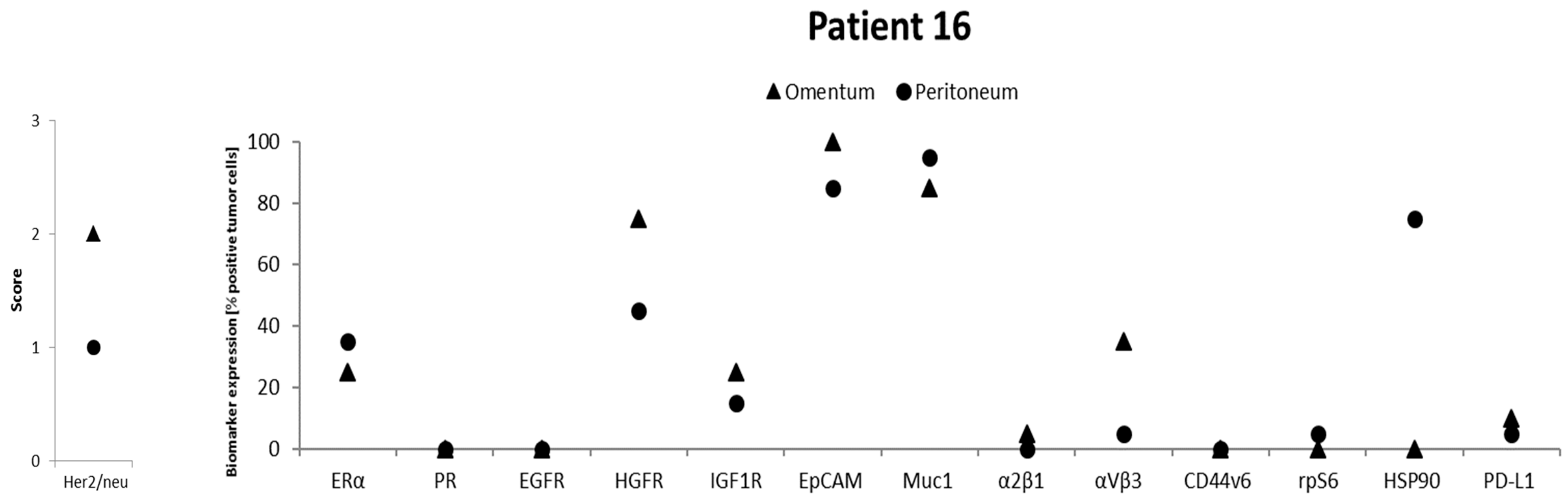

(e)

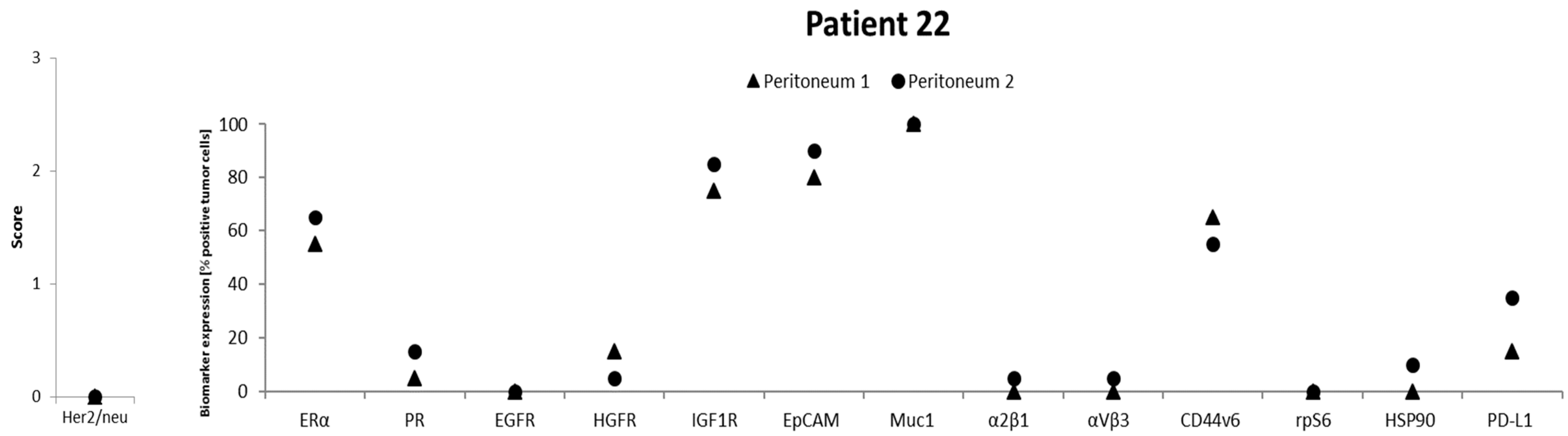

(f)

Figure S2. Intra-patient heterogeneity of drugable biomarker expression analyzed in recurrent ovarian cancers.

Figure S3:

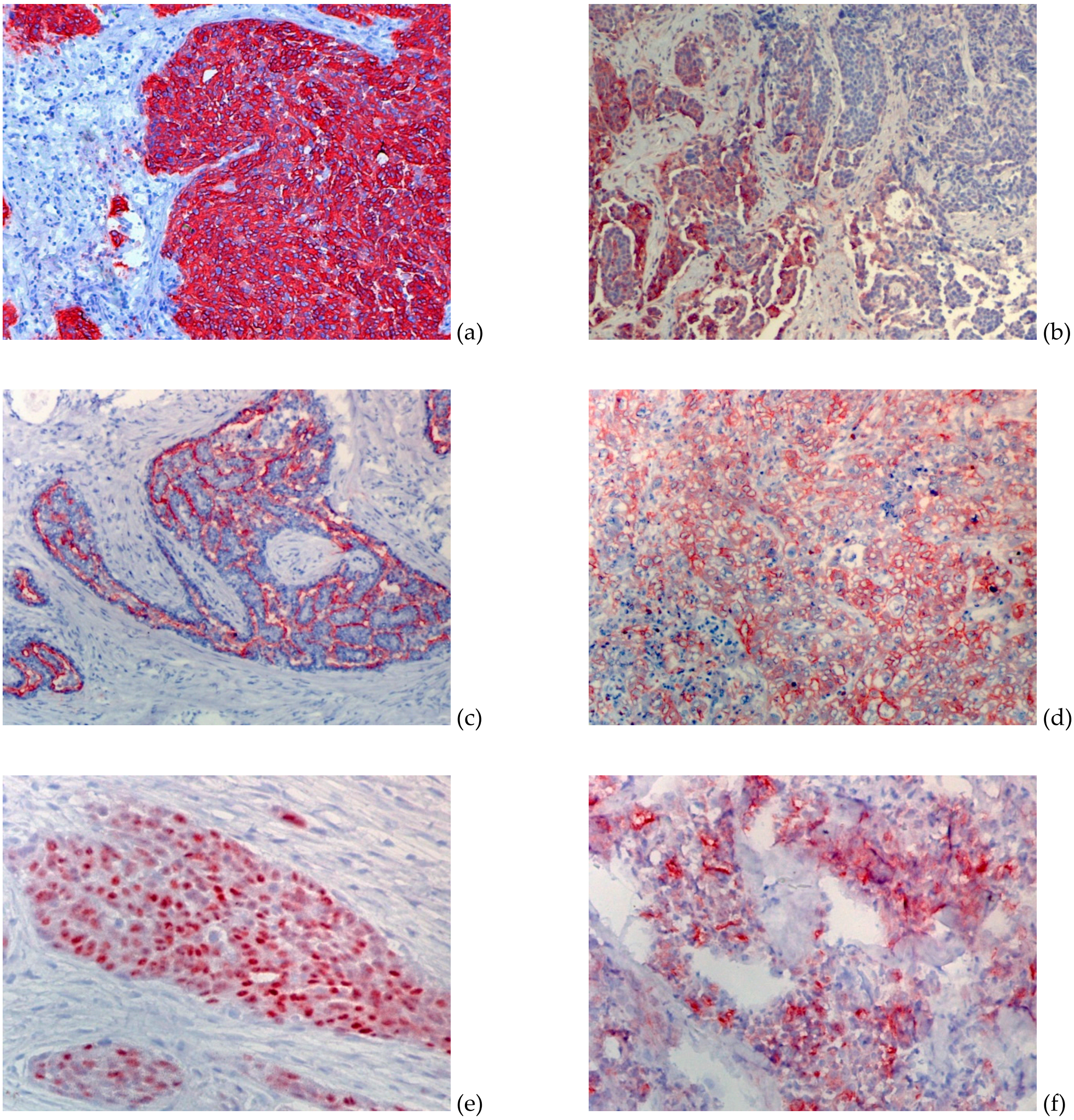

Figure S3. Immunohistochemical staining of different biomarkers in recurrent ovarian cancer **(a)** Staining of EpCAM of Patient 9 (100x magnification), **(b)** Staining of rpS6 of Patient 21 (100x magnification), **(c)** Staining of MUC1 of Patient 17 (100x magnification), **(d)** Staining of CD44v6 of Patient 13 (100x magnification), **(e)** Staining of ERα of Patient 11 (200x magnification), **(f)** Staining of Her2/neu of Patient 7 (200x magnification)

Figure S4:

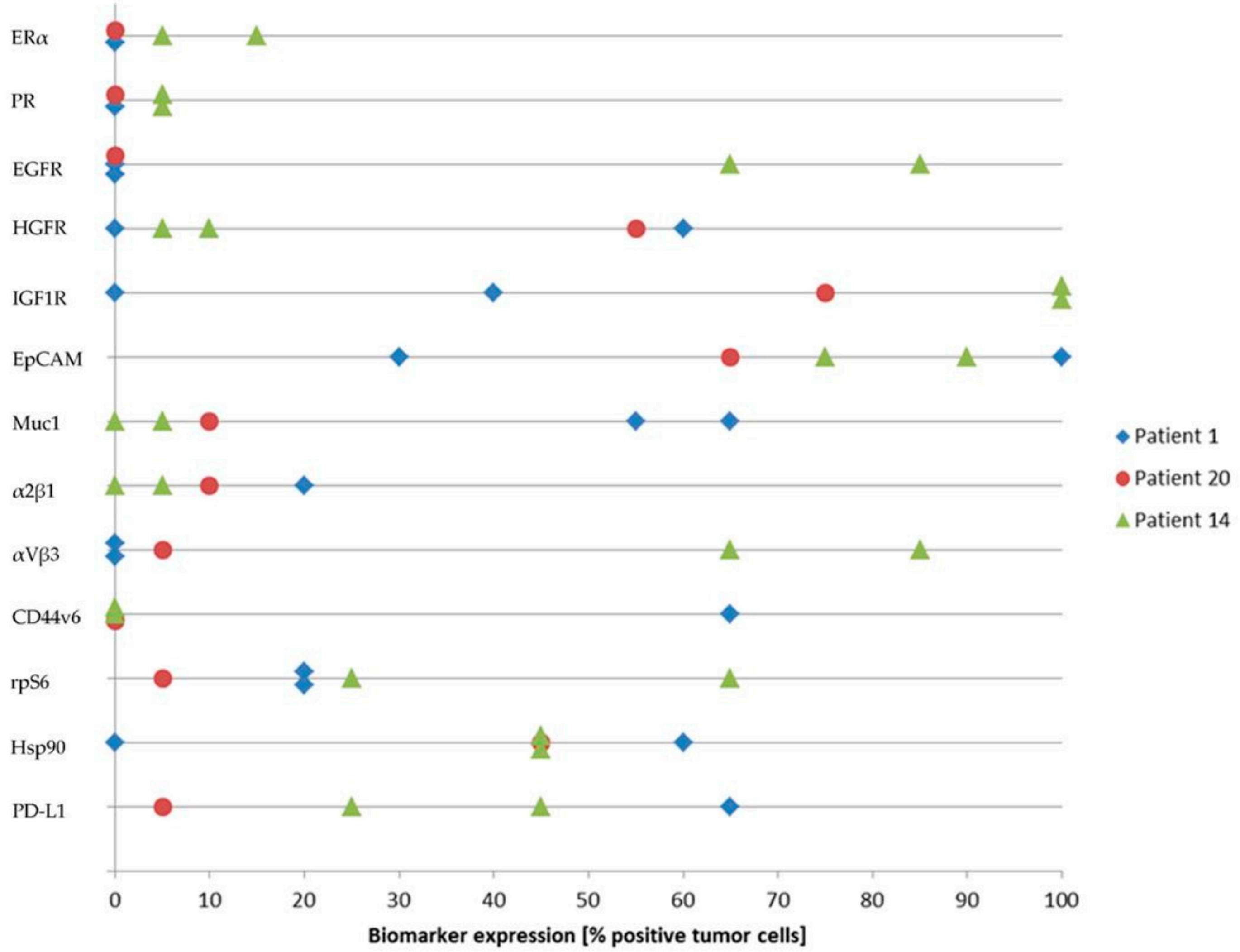

Figure S4. Immunohistochemical biomarker profiling in recurrent non-HGSOC ovarian cancer. Each symbol represents a tumor sample. Evaluation criteria are described in Material and Methods.
